# Attentional Bias in Snus Users: An Experimental Study

**DOI:** 10.1371/journal.pone.0108897

**Published:** 2014-10-08

**Authors:** Rune Aune Mentzoni, Bjørn Sætrevik, Helge Molde, Nora Wiium, Jørn Hetland, Ida Fagerland, Linn Tinnesand Nordnes, Sunniva Straume Storemark, Ingrid Nesdal Fossum, Ståle Pallesen

**Affiliations:** 1 Department of Psychosocial Science, Faculty of Psychology, University of Bergen, Bergen, Norway; 2 Department of Clinical Psychology, Faculty of Psychology, University of Bergen, Bergen, Norway; Universidad de Granada, Spain

## Abstract

The use of nicotine in the form of “snus” is substantial and increasing in some geographic areas, in particular among young people. It has previously been suggested that addictions may operate through a mechanism of attentional bias, in which stimuli representative of the dependent substance increase in salience, thus increasing the addictive behavior. However, this hypothesis has not been tested for the case of snus. The current experiment used a modified Stroop task and a dot-probe task to investigate whether 40 snus users show an attentional bias towards snus-relevant stimuli, compared to 40 non-snus users. There were no significant differences between the two groups on reaction times or accuracy on either Stroop or dot-probe task, thus failing to show an attentional bias towards snus-relevant stimuli for snus users. This could imply that other mechanisms may contribute to maintenance of snus use than for other addictions. However, this is the first experimental study investigating attentional bias in snus users, and more research is warranted.

## Introduction

During the past few decades there has been an increase in the use of smokeless tobacco, including moist snuff (snus) [1]. Its use is a relatively restricted phenomenon in terms of geographical range, with the prevalence being particularly high in Norway and Sweden [2]. However, snus use extends beyond Scandinavia, and increased use has also been observed in the USA [1]. In Norway, 9% of the population used snus on a daily basis in 2013, while 4% used it occasionally [3]. The use is most frequent in the younger population, in which 19% of young adults between the ages of 16 and 24 reported daily snus use [3]. An increase in snus use was observed after the introduction of strict regulations on smoking [4]. Like cigarettes and other tobacco products, snus contains nicotine, and its addictive potential has been widely recognized [5]–[7].

The use of smokeless tobacco such as snus may be associated with an increased risk of various adverse health consequences such as oral mucosal lesions, periodontal and gingival diseases, tooth loss [8], fatal myocardial infarction [9], [10] and pancreatic cancer [11], [12]. However, the evidence is conflicting [1], [8], and some studies have failed to identify negative health consequences [13], [14]. Nevertheless, addiction to nicotine might be considered a problem in itself [15].

A vast amount of research has been conducted with the aim of identifying cognitive factors underlying substance use and addiction in general. According to the cognitive processing model of Tiffany [16], addiction and substance use is mainly controlled by automatic or implicit processes. Rooke, Hine and Thorsteinsson [17] highlight attentional bias as a facet of implicit cognition that may influence decisions and behaviors that regulate substance use. Attentional bias can be defined as modified attentional processing of addiction-relevant stimuli [18]. Through past learning experiences with a particular substance, substance cues in the environment may gain incentive value and thereby be perceived as highly attractive. These cues may as a result automatically capture attention, thereby exerting influence on behavior [17]. In cases where substance use is prevented, the person may experience increased substance-urge and craving. Craving is assumed to be accompanied by an attentional bias towards substances-related cues [16]. Hence, craving and urge to use a substance can enhance attentional bias for the substance-related cues [19]. Consequently, there is reason to believe that attentional bias may contribute to maintenance of substance-use behavior and addiction.

Attentional bias has been demonstrated in users of several different substances such as smoking tobacco [20]–[22], alcohol [23]–[29], cocaine [30] and opiates [31], [32]. Attentional bias has also been investigated in relation to problematic gambling, with the findings generally consistent with those concerning substance use [18], [33].

In order to study substance-related attentional bias in substance users, both direct and indirect measures have been used [34]. When using indirect measures, attentional processing is inferred from increased or decreased reaction time (RT) to addiction-related stimuli compared to neutral stimuli [18]. Two of the most widely used indirect measures comprise the Stroop task and the dot-probe (visual probe) task.

In the classical Stroop task, participants are presented with color words (i.e., names of colors) in different colored print. Word meaning and ink color are either congruent (e.g. the word “blue” printed in blue letters) or incongruent (e.g. the word “blue” printed in red letters). Participants are instructed to name the print color while ignoring the meaning of the word. The Stroop effect refers to the finding that participants are slower to name the correct color in incongruent trials, compared to congruent trials [35]. In the addiction Stroop task, participants are presented with neutral or addiction-related words and/or pictures. The words are written in differently colored print, while the pictures are presented within colored frames [36]. The participants are asked to name the color the word is printed in or name the color of the frame, while ignoring the meaning of the words or the pictures. If participants show longer RTs to addiction-related stimuli than to neutral stimuli, it is assumed that the task-irrelevant word or picture meaning interferes with the task-relevant color processing, indicating an attentional bias towards the addiction-related stimuli [36].

In the dot-probe task, the participants are presented with an addiction-related stimulus (a picture or a word) and a matched control stimulus simultaneously on a computer screen [34]. The stimuli disappear, and a probe or cue replaces either one of the two stimuli. The participants' RT to the probes is measured. Attention is assumed primarily to be drawn to location where the addiction-related stimulus was present. Attentional bias is indicated when RT to probes that replace addiction-related stimuli are faster than RT to probes replacing neutral stimuli [34]. Attentional bias towards addiction-related stimuli has previously been demonstrated both by using word and pictorial Stroop tasks [22], [30], as well as word and pictorial dot-probe tasks [21], [25].

As previous studies have demonstrated attentional bias in various addictions, it is of interest to investigate whether such a bias exists in relation to snus use as well. To the best of our knowledge no such study has yet been conducted. Gaining knowledge about the presence or absence of attentional bias in snus users will contribute to the understanding of factors that maintain snus use. This is important since snus use is in rapid growth and may be associated with adverse health consequences. Furthermore, attentional bias among snus users might have implications for treatment approaches [37].

Against this backdrop we conducted an experiment where Stroop and dot-probe tasks were used to investigate whether snus users show attentional bias towards snus-related stimuli. We hypothesized that snus users would show attentional bias towards snus-related stimuli. Concerning the Stroop task, we specifically expected that, compared to control participants, snus users' color naming on snus-related stimuli would be relatively slower than for neutral stimuli. In the dot-probe task we specifically expected that, compared to control participants, snus users would have relatively faster responses when the probe replaced snus-related stimuli than when the probe replaced neutral stimuli. We expected that attentional bias would be evident in both pictorial and word stimuli conditions across both tasks.

## Methods

Participants were informed about the study in lectures or on Facebook, and provided written consent to be contacted for future participation in the experiment. They were then contacted by phone and provided verbal consent to participate on a specific date and time. The study was conducted in accordance with the principles expressed in the Declaration of Helsinki, and the Regional Committee for Medical Research Ethics deemed the procedure not necessary to submit for approval. The Research Committee at the Faculty of Psychology, University of Bergen, approved the ethical aspects of the study.

### Sample

The sample consisted of 80 young adults aged 18–28. Sixty participants were recruited based on the responses on a questionnaire measuring nicotine habits that was distributed in undergraduate psychology lectures at the University of Bergen, and twenty participants were recruited through “peer-to-peer” advertisement on Facebook. The snus group consisted of 40 snus-users who had used snus on a daily basis during the last three months (mean age = 21.8, *SD* = 2.34, mean number of snus doses per day = 10.5, *SD* = 5.0). In this group, 82.5% (*n* = 33) reported to have used snus within the hour before testing, 5% (*n* = 2) had used snus within 3 hours prior to testing, whereas 12.5% (*n* = 5) had not yet used snus on the day of testing. The control group consisted of 40 participants who had not smoked cigarettes or used snus during the last three months, and who had not used nicotine or snus on a daily basis during their lifetime (mean age = 21.1, *SD* = 2.68). Both groups consisted of 20 males and 20 females. A self-report questionnaire was completed on arrival for verification that inclusion criteria were met. Six controls no longer fulfilled the inclusion criteria concerning nicotine abstinence during the last three months; three reported smoking occasionally and three reported having used snus at least once within this timeframe. These participants were consequently excluded from all analyses. Four controls did not report whether or not they had smoked cigarettes or used snus during their lifetime. These were included in the analyses. All participants reported normal color vision.

### Design

#### Stroop task

A 2×2×2 repeated measures analysis of variance (ANOVA) was conducted with the Stroop task data, with group (snus users/controls) as between subjects-factor. Stimuli mode (words/pictures) and stimuli type (relevant/neutral) were within-subjects factors. Dependent variables were color-naming RT from the word or picture onset to registered key-press, as well as accuracy.

#### Dot-probe task

A 2×2×2 repeated measures ANOVA was conducted with the dot-probe task data with group (snus-users/controls) as between-subjects factor. Stimuli mode (words/pictures) and stimuli type (relevant/neutral) were within-subjects factors. Dependent variables were RT from probe onset to registered key-press, and accuracy.

### Procedure, Apparatus and Materials

The experiment was performed in a multiple testing lab with fully enclosed cubicles allowing for simultaneous testing of up to five participants. All participants conducted the Stroop task and the dot-probe task. The order of the tasks was counterbalanced between participants. Snus use was not allowed during testing. The entire experiment lasted for approximately 30 minutes, and participants received 50 NOK (9 USD) for their participation.

The Stroop and dot-probe tasks were programmed and run in E-prime 2. The experiments were conducted on desktop computers with a 19″ CRT monitor with a resolution of 1024*768 and a refresh rate of 85 Hz. In both tasks, responses were given on a standard QWERTY keyboard.

#### Stroop Task

A modified version of the addiction Stroop task was used. The task was divided into two parts, one part using picture stimuli and the other using word stimuli. Four adjacent keys (F, G, H, and J) on the keyboard had been marked as response buttons with white stickers. The keys corresponded to four adjacent boxes shown on screen throughout the task, containing the relevant color names (red, green, blue and yellow) written in black. The stimuli were presented in sixteen blocks of eight trials, in which eight blocks contained text stimuli and eight contained picture stimuli, constituting a total of 128 trials. Blocks of pictures and words were presented every other time, with half of the participants starting with pictures and the other half with words. The presentation software counterbalanced the order of stimuli blocks. The order of stimuli presentation within each block was randomized across participants. Before the stimuli were presented, a fixation point appeared on the screen for 1000 ms. The stimuli were then presented for 3000 ms, or until the participant responded. The inter-trial interval (a blank screen) was either 750, 1000 or 1250 ms, selected at random by the software. Six training trials were presented while under supervision from a researcher prior to the main task in order to familiarize participants with the response mapping. The training task differed from the experimental task in that participants only responded to the color of simple squares. The stimuli were presented with the same time intervals as in the experiment.

#### Word addiction Stroop task

The word list (see Table S1 in File S1) was prepared by the authors for the purpose of the present study, and included 50 words (25 relevant and 25 neutral). The neutral and relevant words were matched for number of syllables. The words were presented individually in font Arial and font size 24 in the middle of the computer screen. Each word was lettered in one of four basic colors (selection on-line randomized), red (RGB code 255, 0, 0), blue (RGB code 0, 0, 255), green (RGB code 0, 128, 0) or yellow (RGB code 255, 255, 0) on a silver background (RGB code 192, 192, 192). Participants indicated the color of the word by pressing one of the four different response buttons.

#### Pictorial addiction Stroop task

The picture list (see Figure S1 in File S1 for examples of the stimuli) used was also prepared for the purpose of this study, and included 50 photographs (25 relevant and 25 neutral). All pictures displayed centrally placed objects on a white background. The relevant pictures showed various snus products. The neutral pictures showed various office tools and appliances. The pictures were presented individually in the middle of the computer screen within colored frames in red, blue, green or yellow. The pictures had a resolution of 307*230, and the frames were 20 pixels broad in each direction. The participants indicated the color of the frame by pressing one of the four response buttons.

#### Dot-probe task

The dot-probe task included 100 trials, each trial consisting of two stimuli. Fifty trials consisted of picture stimuli, and 50 trials consisted of word stimuli. The software randomized for each trial whether pictures or words were presented. The participants were presented with either two words or two pictures simultaneously on the computer screen, one above the other. There were three trial types, 1) mixed, with one neutral and one relevant stimuli (50 trials, half of which presented the relevant stimuli in the top position, half in the bottom position. Order was randomized), 2) neutral stimuli in both positions (25 trials) and 3) relevant stimuli in both positions (25 trials). Before the stimuli were presented, a fixation point (“+”) appeared in the middle of the screen for 1000 ms. Next, two stimuli were presented for 750 ms. A probe (O) was then introduced as a replacement for one of the stimuli, and the participants were instructed to indicate the position of the probe. The probe was presented for 3000 ms, or until the participants responded by pressing one of the arrow keys at the bottom right of the keyboard. The inter-trial interval (a blank screen) was either 750, 1000 or 1250 ms, selected at random by the software.

#### Word dot-probe task

The word list was identical to the word addiction Stroop task. The words were presented simultaneously on the computer screen, one above the other, written in black on a white background. The participants indicated the position of the probe by pressing one of the two arrow keys.

#### Pictorial dot-probe task

The picture list was identical to the pictorial addiction Stroop task. Two pictures were presented simultaneously on the computer screen, one above the other, on a white background. The pictures had a resolution of 307*230. The participants indicated the position of the probe by pressing one of the two arrow keys.

## Results

### Stroop Task

#### Outliers

Prior to the analysis of the Stroop task, we identified outliers with mean RTs exceeding the cut-off value set to ± three standard deviations from the mean. Seven outliers with RTs above the cut-off value were identified and replaced by the mean value plus three standard deviations.

#### Reaction Time

Table 1 shows descriptive statistics for the mean RTs of the two groups across stimuli mode and stimuli type. The mean RTs were analyzed using a 2×2×2 repeated measures ANOVA with stimuli mode (pictures/words) and stimuli type (relevant/neutral) as the two within-subjects factors, and with group as the between-subjects factor. There were no significant main effects for group, *F*(1,72) = 0.260, *p* = .61, stimuli type, *F*(1,72) = 0.580, *p* = .45, or stimuli mode, *F*(1,72) = 2.579, *p* = .11. Further, neither the stimuli mode by stimuli type interaction, *F*(1,72) = 1.803, *p* = .18, nor the group by stimuli mode interaction, *F*(1,72) = 0.478, *p* = .49, was significant. In addition, there was no significant interaction between group membership and stimuli type, *F*(1,72) = 0.023, *p* = .88, and no significant three-way interaction for group, mode and stimuli type, *F*(1,72) = 0.025, *p* = .88. There were no differences in RTs between the groups across stimuli mode and stimuli type.

**Table 1:** Reaction Time Across Stimuli Type and Stimuli Mode in Stroop task.

	Control			Snus		
Mode/Type	M	SD	N	M	SD	N
Picture/Relevant	812.26	183.71	34	829.98	201.63	40
Picture/Neutral	813.91	183.88	34	832.08	199.02	40
Word/Relevant	805.94	180.43	34	830.69	215.13	40
Word/Neutral	791.09	151.77	34	819.76	214.04	40

#### Accuracy

Table 2 shows descriptive statistics for accuracy performance across stimuli type and stimuli mode. Accuracy (proportion of correct responses) was analyzed using a 2×2×2 repeated measures ANOVA. The results showed no significant differences in accuracy between the snus and control group, *F*(1,72) = .010, *p* = .92. The three-way interaction between group, mode and stimuli type was not significant, *F*(1,72) = .431, *p* = .51. Accuracy performance for both snus users and controls was close to ceiling (over 95%) for both stimuli mode and stimuli type.

**Table 2:** Accuracy Across Mode and Stimuli Type for Stroop Task.

	Control			Snus		
Mode/Type	M	SD	N	M	SD	N
Picture/Relevant	.97	.03	34	.97	.03	40
Picture/Neutral	.97	.03	34	.97	.03	40
Word/Relevant	.98	.03	34	.97	.02	40
Word/Neutral	.97	.03	34	.97	.03	40

### Dot-Probe Task

#### Outliers

Before conducting the analysis, we identified outliers with mean RTs exceeding the ± three standard deviations from the mean. Two outliers with RTs above the cut off value were identified. In these cases, RTs were replaced by the mean plus three standard deviations. Three participants from the control group were removed from the analysis because no correct responses were recorded.

#### Reaction Time

Table 3 shows descriptive statistics for the mean RTs of the two groups across mode and stimuli type. The mean RTs for dot-probe were analyzed using a 2×2×2 repeated measures ANOVA with stimuli type (relevant/neutral) and stimuli mode (pictures/words) as within-subjects factors, and with group (snus users/controls) as the between-subjects factor. The results showed no significant main effect for group, *F*(1,69) = 0.024, *p* = .88. Nor were there any significant main effect for stimuli type, *F*(1,69) = 0.733, *p* = .40. However, there was a significant main effect for stimuli mode, *F*(1,69) = 10,467, *p*<.01, in the direction of faster RTs for word stimuli. Neither the stimuli mode by stimuli type interaction, *F*(1,69) = 0.198, *p* = .66, nor the group membership by mode interaction was significant, *F*(1,69) = 0.806, *p* = 0.37. There were no significant interaction between group and stimuli type, *F*(1,69) = 0.003, *p* = 0.96, and no significant three-way interaction for group, mode and stimuli type, *F*(1,69) = 0.524, *p* = .47. There were no differences in RTs between the groups across stimuli mode and stimuli type

**Table 3:** Reaction Time Across Mode and Stimuli Type for Dot-Probe Task.

	Control			Snus		
Mode/Type	M	SD	N	M	SD	N
Picture/Relevant	480.83	86.09	31	476.31	80.48	40
Picture/Neutral	471.09	75.63	31	473.47	88.38	40
Word/Relevant	459.82	78.77	31	468.03	68.01	40
Word/Neutral	459.46	77.61	31	462.73	63.69	40

#### Accuracy

Table 4 shows descriptive statistics for accuracy performance across stimuli type and stimuli mode. Accuracy was analyzed using a 2×2×2 repeated measures ANOVA. The results showed no significant differences in accuracy between the snus and control group, F(1,69) = 2.216, *p* = .14. Nor was the three-way interaction between group, mode and stimuli type significant, F(1,69) = .527, *p* = .47. Accuracy performance for both snus users and controls was close to ceiling values (over 95%) for both stimuli mode and stimuli type.

**Table 4:** Accuracy Across Mode and Stimuli Type for Dot-probe Task.

	Control			Snus		
Mode/Type	M	SD	N	M	SD	N
Picture/Relevant	.99	.03	31	.99	.03	40
Picture/Neutral	.99	.02	31	1.00	.02	40
Word/Relevant	.98	.04	31	.99	.02	40
Word/Neutral	.98	.04	31	.99	.02	40

## Discussion

The present study's main aim was to investigate attentional bias towards snus-related stimuli among snus users. Based on a review of the literature concerning substance use and behavioral addictions, we hypothesized that snus users would show attentional bias towards snus-related stimuli, and that this effect would be evident in both the Stroop and dot-probe tasks. The results from the Stroop task and dot-probe task showed that snus users did not differ from the controls in terms of RT's to snus-related stimuli and neutral stimuli and the Group x Stimuli interaction was not significant. These findings applied to word as well as to pictorial stimuli. Thus the results from the current experiment did not support our predictions of an attentional bias for snus users towards snus-relevant stimuli. The absence of attentional bias was mirrored in terms of accuracy. There were no differences between the two groups, with both groups' accuracy performance being over 95%. There was no observed difference in accuracy between groups or across conditions.

No study on attentional bias towards snus-stimuli has previously been reported. However, our results were inconsistent with the majority of previous research on related addictions, where attentional bias has been demonstrated across different types of addictions such as alcohol use, smoking and gambling [18], [34], [36].

One possible explanation for why attentional bias could not be demonstrated in the present study could be that some of the snus-stimuli were brand specific. Thus, the snus-stimuli's potential for eliciting attentional bias may have varied across subjects depending on their brand preferences. However, because many of the snus-stimuli used in present study were of a generic type, particularly the words, the snus-related stimuli should not be too idiosyncratic in terms of their overall attentional bias potential. Also, the majority of previous studies demonstrating an attentional bias in related addictions have used generalized addiction-related stimuli and one study failed to find any special impact of personalized stimuli over and above the influence of generalized alcohol-related stimuli [38].

With a mean daily snus intake of 10.5 doses, the sample in our study consisted predominantly of what would be considered as moderate to heavy snus users. However, it cannot be ruled out that a sample consisting of more extreme users would give different results. Since the magnitude of use have been linked to attentional bias towards several other types of substances [34], studies investigating extreme users of snus would be a welcome addition to the field. An alternative explanation for why snus users did not show an attentional bias can be derived from Tiffany's model [16]. When applied to snus use, this model suggests that craving for snus will cause attentional bias towards snus-related stimuli. However, snus is easily obtainable and can, in contrast to narcotics, cigarettes and alcohol, be openly administered in almost all contexts, without raising concerns or objections. In line with this, there is reason to assume that snus users will experience low levels of craving and urges compared to users of other substances. Consequently, it is possible that snus use in general does not induce the levels of craving needed to elicit attentional bias, which would explain our findings. If this assumption is correct attentional bias should be evident if snus-users were kept abstinent for some time [19] and future studies should investigate this possibility.

### Strengths and Limitations

No measure of craving was employed in our study. However, since craving is assumed to increase attentional bias [34], a measure of this aspect would have been a useful study variable. Future studies on attentional bias related to snus and other addictions should therefore include such measure. In addition, the participants of the current study were not asked to provide information about use of other substances than smoking and snus. Theoretically, it could be the case that use of other drugs could influence the results. Future studies should therefore carefully assess and control for substances beside the one targeted for study.

The current study's Stroop task was conducted on a computer with keyboard responses. Some studies have indicated that presenting the Stroop stimuli on cards with vocal responses provides stronger Stroop interference [39]. However, several studies have demonstrated attentional bias towards addiction-related stimuli by conducting the Stroop task on a computer [22], [26], [31], [33], therefore it is reasonable to assume that this procedure should reveal an attentional bias in snus users if it was present.

A potential asset of the study is that it included two separate measures of attentional bias, Stroop task and dot-probe. This provides broader information about attentional processes in snus users than when using either of these tests alone. As an additional asset, both tests include pictorial and word stimuli, providing a greater likelihood to reveal a potential attentional bias. Since both pictorial and word conditions in the two tests provided similar results, the inferences that can be drawn from the obtained results are strengthened.

## Conclusions

Our findings did not support the hypothesis that snus users would show an attentional bias towards snus-related stimuli. However, this is the first study to investigate attentional bias in snus users, and more research is needed in order to determine whether such an attentional bias is present in snus users or not. If future studies also fail to show snus users to have attentional bias towards snus-related stimuli, this would suggest that attentional processes in snus addiction differ from attentional processes in other addictions. It is therefore important that future research attempts to identify other potential underlying mechanisms of snus addiction.

More research is warranted to identify the processes underlying snus use and addiction. It is especially important to gain such knowledge since there has been an increase in use of snus in the recent years and because of the potential adverse health consequences associated with snus use. Knowledge about underlying factors could expand the understanding of snus use and addiction, and may have implications for treatment of snus addiction.

## Supporting Information

File S1 **This file contains additional information for this article and includes Table S1 and Figure S1.** Table S1, List of neutral and relevant words used in the experiment. Figure S1, Example of relevant and neutral picture used in the experiment (logo and brand names were not blurred).
(DOCX)
