# Supplementary material for: Attentional Bias in Snus Users: An Experimental Study
Source: PLoS One. 2014 Oct 8;9(10):e108897. doi: 10.1371/journal.pone.0108897 (PMC4189952; doi:10.1371/journal.pone.0108897)

Table S1

| Neutral Words | Relevant Words |
| --- | --- |
| Teppe (rug) | Snus (snus) |
| Teppene (rugs) | Snusen (the snus) |
| Tenker (thinks) | Snuser (to take snus) |
| Tenkte (thought) | Snuste (has taken snus) |
| Tenkt (has thought) | Snust (has taken snus) |
| Landsby (village) | Snusboks (a box of snus) |
| Landsbyen (the village) | Snusboksen (the box of snus) |
| Landsbyer (villages) | Snusbokser (boxes of snus) |
| Landsbyene (the villages) | Snusboksene (the boxes of snus) |
| Frontrute (windshield) | Snusleppe (a snus user’s lip) |
| Frontruten (the windshield) | Snusleppen (the snus user’s lip) |
| Frontruter (headlights) | Snuslepper (a snus user’s lips) |
| Frontrutene (the headlights) | Snusleppene (the snus user’s lips) |
| Kjøpesenter (shopping mall) | Porsjonssnus (portion snus) |
| Kjøpesenteret (the shopping mall) | Porsjonssnusen (the portion snus) |
| Kjøpesentre (shopping malls) | Porsjonssnuser (to take portion snus) |
| Kjøpesentrene (the shopping malls) | Porsjonssnusene (the portion snuses) |
| Bokreol (book case) | General (snus brand name) |
| Benk (bench) | Skruf (snus brand name) |
| Glass (glass) | Catch (snus brand name) |
| Høytaler (loudspeaker) | Snusdåse (synonym to box of snus) |
| Høytaleren (the loudspeaker) | Snusdåsen (box of snus) |
| Høytalere (loudspeakers) | Snusdåser (boxes of snus) |
| Høytalerne (the loudspeakers) | Snusdåsene (the boxes of snus) |
| Innhold (content) | Løssnus (snus that is not portion snus) |

Figure S1

Example of snus-relevant picture (logo and brand names were not blurred):


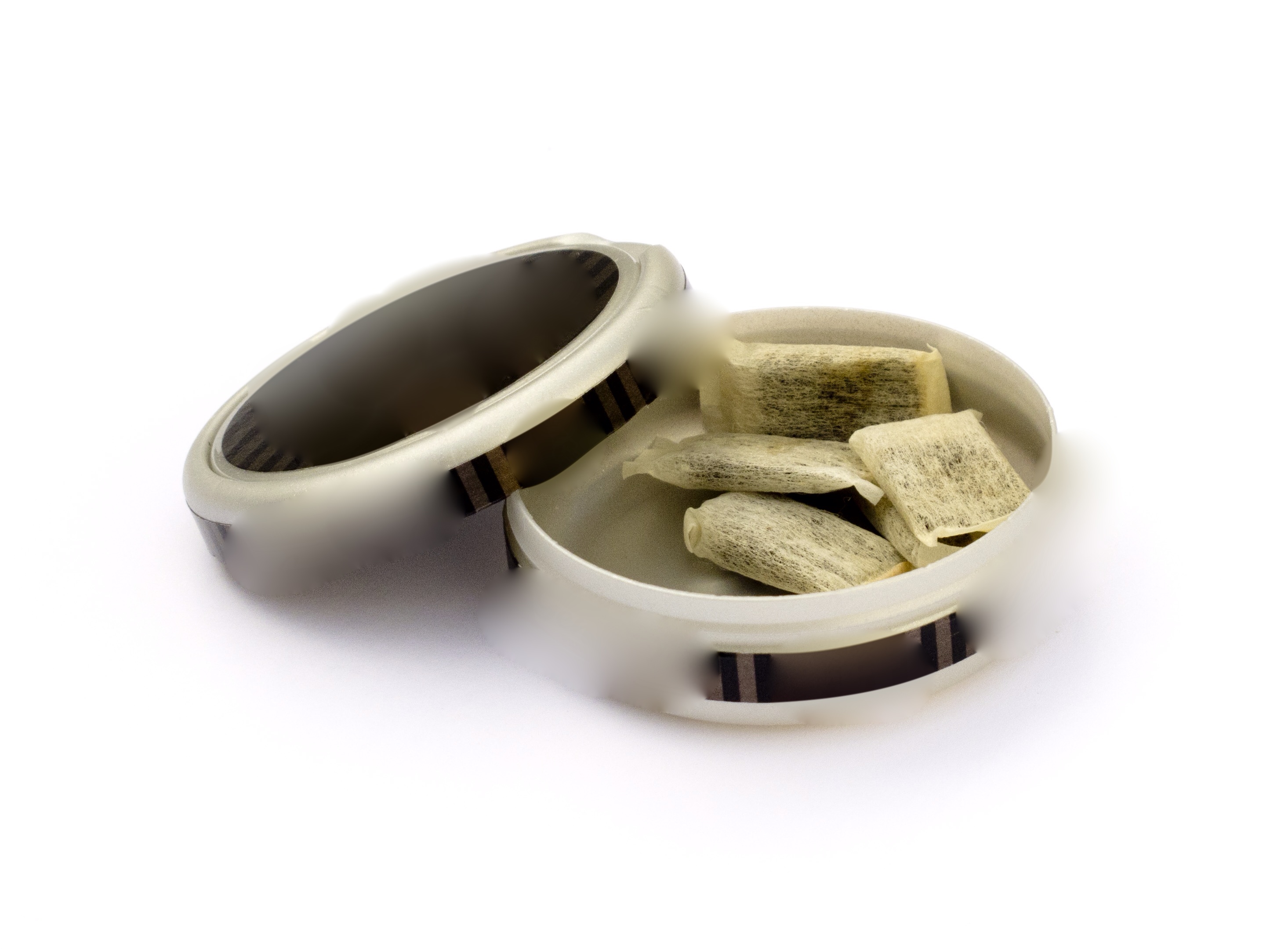


Example of neutral picture (logo and brand names were not blurred):


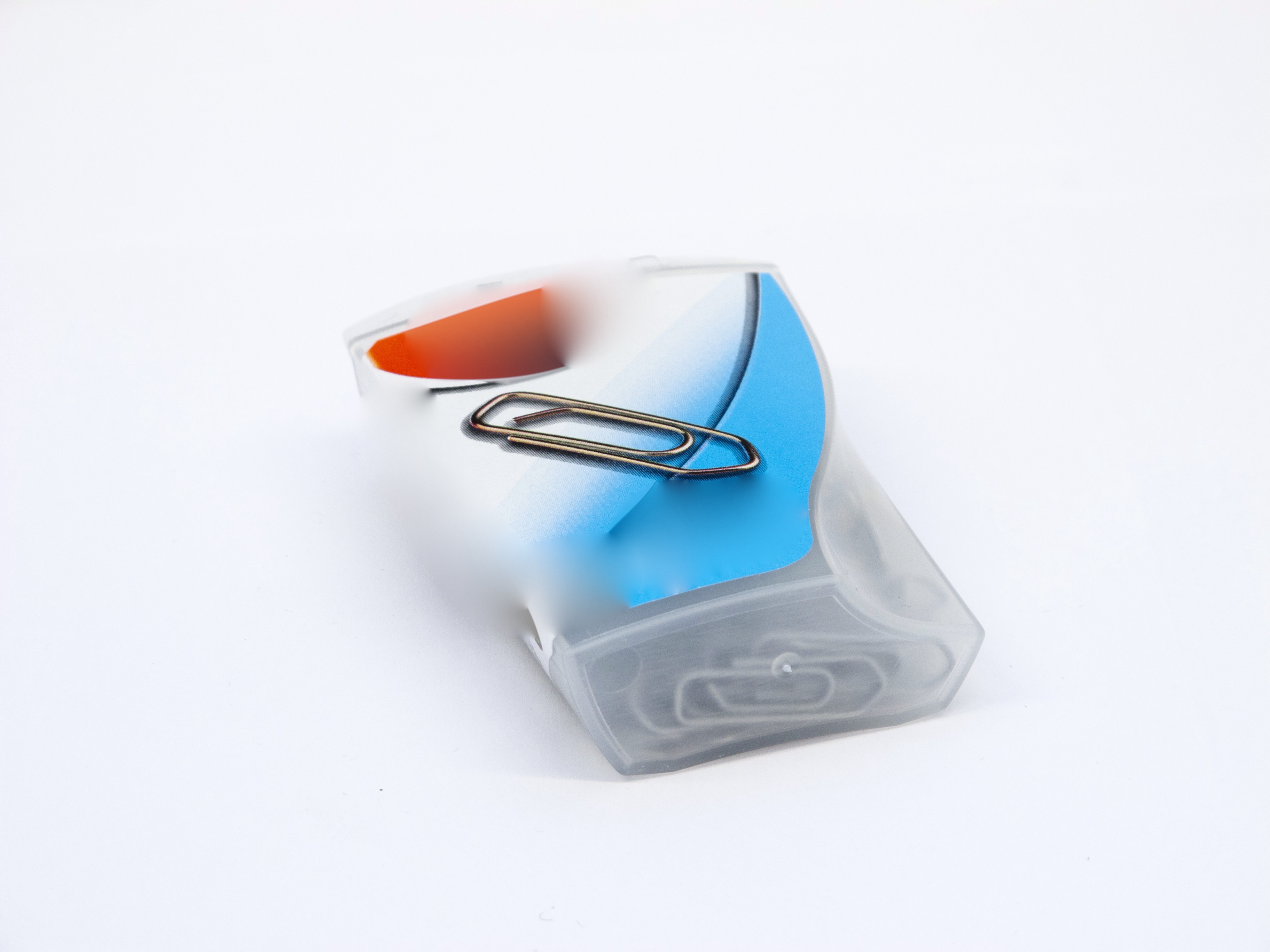

Supplement: File S1 — This file contains additional information for this article and includes Table S1 and Figure S1. Table S1, List of neutral and relevant words used in the experiment. Figure S1, Example of relevant and neutral picture used in the experiment (logo and brand names were not blurred). (DOCX) [file pone.0108897.s001.docx]
